# GPs’ awareness of pregnancy: trends and association with hazardous medication use

**DOI:** 10.3399/BJGP.2022.0193

**Published:** 2023-01-24

**Authors:** Eline Houben, Karin MA Swart, Eric AP Steegers, Petra JM Elders, Ron MC Herings

**Affiliations:** PHARMO Institute for Drug Outcomes Research, Utrecht; Department of Obstetrics and Gynaecology, Erasmus MC, Rotterdam.; PHARMO Institute for Drug Outcomes Research, Utrecht.; Department of Obstetrics and Gynaecology, Erasmus MC, Rotterdam.; Department of General Practice, Amsterdam UMC, Location Vrije Universiteit, Amsterdam Public Health Research Institute, Amsterdam UMC, Amsterdam.; PHARMO Institute for Drug Outcomes Research, Utrecht; Department of Epidemiology and Data Science, Amsterdam Public Health Research Institute, Amsterdam UMC, Amsterdam.

**Keywords:** awareness, electronic health records, general practice, inappropriate prescribing, pregnancy, prescriptions

## Abstract

**Background:**

GPs have been shown to be important providers of medical care during pregnancy, however, little evidence exists on their awareness of pregnancy when prescribing medication to women.

**Aim:**

To assess GPs’ awareness of pregnancy and its association with prescribing medication with potential safety risks.

**Design and setting:**

Population-based study using confirmed pregnancy records linked to GP records from the PHARMO Perinatal Research Network.

**Method:**

GPs’ awareness of pregnancy, defined as the presence of a pregnancy confirmation in the GP information system during pregnancy, was assessed from 2004 to 2020. GP prescriptions of medication with potential safety risks were selected during pregnancy and its association with GPs’ awareness of pregnancy was assessed using multivariable logistic regression.

**Results:**

A pregnancy confirmation was present in the GP records for 48% (*n* = 67 496/140 976) of selected pregnancies, increasing from 28% (*n* = 34/121) in 2004 to 63% in 2020 (*n* = 5763/9124). During 3% (*n* = 4489/140 976) of all pregnancies, the GP prescribed highly hazardous medication with teratogenic effects that should have been (temporarily) avoided. Pregnancy was GP confirmed for only 13% (*n* = 585/4489) at the first occurrence of such a prescription. Comparative analyses showed that women without a pregnancy confirmation were 59% more likely to be prescribed this highly hazardous medication (odds ratio [OR] 1.59, 95% confidence interval [CI] = 1.49 to 1.70) compared with those with a confirmed pregnancy.

**Conclusion:**

Results of this study indicate a potential issue with GP awareness about pregnancy status at the time medication with potential safety risks is prescribed. Although pregnancy registration by GPs improved over the years, inadequate use still seems to be made of the available information systems for appropriate drug surveillance.

## INTRODUCTION

The role of GPs in pregnancy care has been an item for debate for some time in Europe.^1^ Although GPs act as gatekeeper to hospital and specialist care in the Netherlands, midwives have the lead in providing pregnancy care. In cases where there is medical or obstetric pathology then responsibility is taken over by an obstetrician.^2^ Currently, only 2%–6% of all GPs still provide obstetric care.^3^ Despite their reduced involvement,^1^^,^^4^ GPs have been shown to be important providers of routine medical care for pregnant women.^3^ For example, they are still responsible for the large majority of drug prescriptions during pregnancy, including medication with potential safety risks, in one-third of Dutch pregnancies.^5^^,^^6^ A substantial part of repeat prescribing by GPs occurs without any direct patient contact, thus without assessing pregnancy status or intention.^7^^,^^8^ This underscores the GPs’ vital role in optimisation of pregnancy care.^9^ Collaboration between GPs and midwives has been widely encouraged and GPs acknowledge their role in shared perinatal care.^10^^–^^14^

In practice the involvement of GPs in this collaborative preconception and antenatal care still needs further reinforcement, for instance by their greater involvement in preconception care.^3^^,^^11^^,^^12^^,^^15^ Guidelines state that midwives should inform the GP about pregnancy,^16^ however, no automatic link exists between the information systems used by midwives and GPs. There is little evidence on actual clinical practice in forwarding, recording, and using information about pregnancy.

This study aimed to fill this evidence gap, to allow for more directed future interventions targeted at preventing use of potentially harmful medication during pregnancy. Therefore, the objectives were to assess GPs’ awareness of pregnancy, the way it is registered in GP records, as well as the trends over time. Furthermore, the association between GPs’ awareness and prescribing medication with potential safety risks was assessed.

## METHOD

### Data source

This population-based study was performed using the PHARMO Perinatal Research Network (PPRN), including linked records from the Netherlands Perinatal Registry (Perined) and the PHARMO Database Network (PHARMO).^17^ Perined is a nationwide registry that contains validated data from pregnancies with a gestational age of ≥16 weeks.^18^ PHARMO is a population- based, patient- level network of healthcare databases linking data from different healthcare settings for approximately 25% of the Dutch population.^19^^–^^21^ For the current study two PHARMO databases linked to Perined were selected: the GP Database, comprising data from electronic patient records registered by GPs, and the Out- patient Pharmacy Database, containing detailed drug information from both GP- and specialist-prescribed prescriptions. Mandatory health insurance and required registration with a GP makes the GP Database representative of the general Dutch population.^22^^,^^23^ The Out-patient Pharmacy Database represents the Dutch population that has picked up prescription drugs or has registered with a pharmacy and has been shown to be representative of the general Dutch population in terms of age and sex.

**Table:** How this fits in

The role of Dutch GPs in pregnancy care has been an item for debate for some time. GPs have been shown to be important providers of medical care during pregnancy, however, little evidence exists on GPs’ awareness of pregnancy when prescribing medication to women. This study indicates a potential issue with GP awareness of pregnancy status at the time medication with potential safety risks is prescribed, placing women and their babies at avoidable risk of exposure to teratogens. Although pregnancy registration by GPs improved over the years, inadequate use still seems to be made of the available information systems for appropriate drug surveillance.

The linkage between PHARMO and Perined has been described in more detail elsewhere (including arrangements for data oversight), but was generally based on the birth date of the mother and child and their addresses.^17^ For the current database research with anonymous data, no ethics committee approval was required.

### Study population

Women who gave birth between 2004 and 2020 were selected from the PPRN. No exclusion criteria were applied to increase the generalisability of the results. Women’s medical details needed to be registered in the selected PHARMO databases from 1 year before the conception date (based on ultrasound or first day of the last menstrual period) until the delivery date as recorded in Perined.

### Characteristics

Characteristics included age at delivery, neighbourhood socioeconomic status (SES),^24^^,^^25^ year of delivery, ethnicity, preconceptional use of medication for chronic conditions (see Supplementary Table S1 for included medication), parity, gestational age, care setting at the start of pregnancy, and birth weight. Furthermore, women’s healthcare utilisation in primary care was assessed in the year before conception as well as during pregnancy, defined by the number of GP visits, GP prescriptions, and incoming specialist letters. The type of electronic GP information system used for holding the maternal medical file was also assessed, as multiple different systems are available for use in GP practices with varying options for registration of patient records in different reference tables.

### GPs’ awareness

The concept of GPs’ awareness of pregnancy was quantified by using all available information from the women’s electronic patient records, considering that this is the information caregivers rely on in daily practice. It was defined at multiple levels of pregnancy indicators recorded in the GP information system:

- Pregnancy confirmation: the presence of a specific coded diagnosis on confirmed pregnancy.
- Pregnancy indicator: the presence of any record indicating that the women is pregnant in all digitally available GP records (for example, an uncoded text note about pregnancy as recorded by the GPs’ assistant after a telephone consultation).
- Pregnancy contraindication: the presence of a recorded pregnancy contraindication in this specific GP reference table, which need to be linked actively and is intended for drug surveillance (that is, without this additional data entry no popup will appear warning about contraindicated medication, even when there is an entry for pregnancy as a diagnostic code).

The timing of pregnancy confirmation was grouped by pregnancy trimester according to the first recorded occurrence. The occurrence of GPs providing formal individual preconception care was assessed by means of a recorded preconceptional GP consultation specifically set up and coded for preconception counselling. Exact underlying definitions and GP reference tables for the defined indicators are detailed in Supplementary Table S2.

### Use of hazardous medication

Use of medication with potential safety risks was determined by means of GP prescriptions recorded or continuing during the pregnancy period. Medication was grouped according to the risk classification system for drugs in pregnancy of the Dutch Teratology Information Service Lareb.^26^ These safety profiles were used to define ‘hazardous medication’ (that is, medication with pharmacological or teratogenic effects that requires monitoring or that should be [temporarily] avoided) as well as ‘highly hazardous medication’ (that is, medication with teratogenic effects that should be [temporarily] avoided). Prescriptions with a recorded pregnancy-driven indication were excluded (for example, progesterone used to try to reduce the risk of preterm birth). Availability of a pregnancy confirmation or pregnancy indicator was assessed at the time the (highly) hazardous medication was first prescribed.

For those women with a pregnancy confirmation, the proportion that used (highly) hazardous medication before and after confirmation was assessed. Sensitivity analyses were performed in which use of the (highly) hazardous medication was defined by medication fills as recorded in the Out-patient Pharmacy Database, which includes both GP- and specialist-prescribed prescriptions. Also, separate analyses were performed categorised by year of delivery (2004–2009, 2010–2014, and 2015–2020).

### Statistical analysis

All analyses were performed using SAS (version 9.4). Logistic regression models were used to calculate odds ratios (ORs) and 95% confidence intervals (CIs) to estimate unadjusted associations between characteristics and pregnancy confirmation. Trends over time for defined GP-recorded pregnancy indicators were tested by Poisson regression (*P*<0.05).

The association between GP-recorded pregnancy indicators and use of hazardous medication was assessed by means of logistic regression analyses, providing ORs and 95% CIs adjusted for age, GP information system, and all other characteristics that remained significant using backward selection (*P*<0.05). Similarly, adjusted logistical models were created to assess the association between pregnancy confirmation and the drug-level use of highly hazardous medication.

## RESULTS

### Study population and characteristics

A total of 140 976 pregnancies among 96 182 women were selected from the PPRN between 2004 and 2020. During 48% (*n* = 67 496) of these pregnancies a pregnancy confirmation was identified in the GP records, indicating GPs’ (potential) awareness of pregnancy. Characteristics of included pregnancies are summarised in Table 1 and stratified by pregnancy confirmation.

**Table 1.** Maternal and obstetric characteristics of included pregnancies, stratified by presence of a GP-recorded pregnancy confirmation

**Characteristic**	**Study cohort (*N* = 140 976)**	**GP-recorded pregnancy confirmation (*n* = 67 496, 48%)**	**No GP-recorded pregnancy confirmation (*n* = 73 480, 52%)**	**With versus without GP-recorded pregnancy confirmation, OR (95% CI)**
**Age at delivery, years**				
≤20, *n* (%)	1532 (1)	526 (1)	1006 (1)	0.57 (0.51 to 0.64)
21–30, *n* (%)	59 350 (42)	28 343 (42)	31 007 (42)	1 (reference)
31–40, *n* (%)	76 629 (54)	37 043 (55)	39 586 (54)	1.02 (1.00 to 1.05)
≥41, *n* (%)	3465 (2)	1584 (2)	1881 (3)	0.92 (0.86 to 0.99)
Mean (SD)	31 (5)	31 (4)	31 (5)	1.03 (1.02 to 1.04)

**Socioeconomic status, *n* (%)**				
Low	38 618 (27)	17 183 (25)	21 435 (29)	0.78 (0.76 to 0.80)
Normal	52 758 (37)	26 734 (40)	26 024 (35)	1 (reference)
High	49 210 (35)	23 449 (35)	25 761 (35)	0.89 (0.86 to 0.91)
Unknown	390 (0.3)	130 (0.2)	260 (0.4)	—

**Year of delivery, *n* (%)**				
2004–2009	19 005 (13)	6606 (10)	12 399 (17)	1 (reference)
2010–2015	63 351 (45)	27 558 (41)	35 793 (49)	1.45 (1.40 to 1.49)
2016–2020	58 620 (42)	33 332 (49)	25 288 (34)	2.47 (2.39 to 2.56)

**Ethnicity,^a^ *n* (%)**				
Caucasian	122 630 (87)	59 233 (88)	63 397 (86)	1 (reference)
Non-Caucasian	16 733 (12)	7534 (11)	9199 (13)	0.88 (0.85 to 0.91)
Unknown	1613 (1)	729 (1)	884 (1)	–

**Preconceptional use of medication for chronic conditions,^b^ *n* (%)**	46 825 (33)	23 816 (35)	23 009 (31)	1.20 (1.17 to 1.22)

**Parity, *n* (%)**				
0	58 771 (42)	28 333 (42)	30 438 (41)	1 (reference)
1	54 431 (39)	25 850 (38)	28 581 (39)	0.97 (0.95 to 0.99)
2	19 543 (14)	9368 (14)	10 175 (14)	0.99 (0.96 to 1.02)
≥3	7731 (5)	3736 (6)	3995 (5)	1.00 (0.96 to 1.05)
Unknown	500 (0.4)	209 (0.3)	291 (0.4)	–

**Gestational age, weeks**				
≤24, *n* (%)	10 064 (7)	2854 (4)	7210 (10)	0.40 (0.39 to 0.42)
25–<28, *n* (%)	352 (0.2)	172 (0.3)	180 (0.2)	0.97 (0.79 to 1.20)
28–<33, *n* (%)	1480 (1)	693 (1)	787 (1)	0.90 (0.81 to 0.99)
33–<37, *n* (%)	7765 (6)	3689 (5)	4076 (6)	0.92 (0.88 to 0.97)
≥37, *n* (%)	121 315 (86)	60 088 (89)	61 227 (83)	1 (reference)
Mean (SD)	38.0 (5.3)	38.5 (4.3)	37.5 (6.0)	1.22 (1.21 to 1.23)^c^

**Care setting at start of pregnancy, *n* (%)**				
Primary care	120 592 (86)	57 685 (85)	62 907 (86)	1 (reference)
Secondary care	20 109 (14)	9694 (14)	10 415 (14)	1.02 (0.99 to 1.05)
Unknown	275 (0.2)	117 (0.2)	158 (0.2)	–

**Birth weight, g, mean (SD)**	3384 (653)	3392 (641)	3377 (665)	1.02 (1.01 to 1.03)^d^

**Healthcare use in primary care**				
Year before conception, mean (SD)				
Number of GP visits	1.2 (2.4)	1.4 (2.5)	1.0 (2.2)	1.09 (1.08 to 1.09)
Number of GP prescriptions	3.9 (4.7)	4.2 (4.8)	3.7 (4.7)	1.02 (1.02 to 1.02)
Number of incoming specialist letters	1.7 (2.2)	1.8 (2.3)	1.5 (2.1)	1.07 (1.07 to 1.08)
During pregnancy, mean (SD)				
Number of GP visits	1.0 (1.9)	1.3 (2.1)	0.8 (1.7)	1.17 (1.16 to 1.18)
Number of GP prescriptions	2.8 (3.7)	3.1 (3.7)	2.5 (3.6)	1.05 (1.04 to 1.05)
Number of incoming specialist letters	1.8 (2.1)	2.3 (2.2)	1.4 (1.9)	1.23 (1.23 to 1.24)

a *Terminology as provided by the database holder.*

b *Based on both GP- and specialist-prescribed medication dispensed in the out-patient pharmacy in the year before conception (see Supplementary Table S1 for included medication).*

c *OR for 5 weeks change.*

d *OR for 500 g change. OR = odds ratio. SD = standard deviation.*

In particular, SES, year of delivery, ethnicity, preconceptional use of medication for chronic conditions, gestational age, and women’s healthcare use in primary care were associated with GP’s being aware of pregnancy. A total of seven different electronic systems were used in the included GP practices. The type of GP information system was found to be significantly associated with GPs’ awareness (data not shown).

### GPs’ awareness

**Figure 1.**
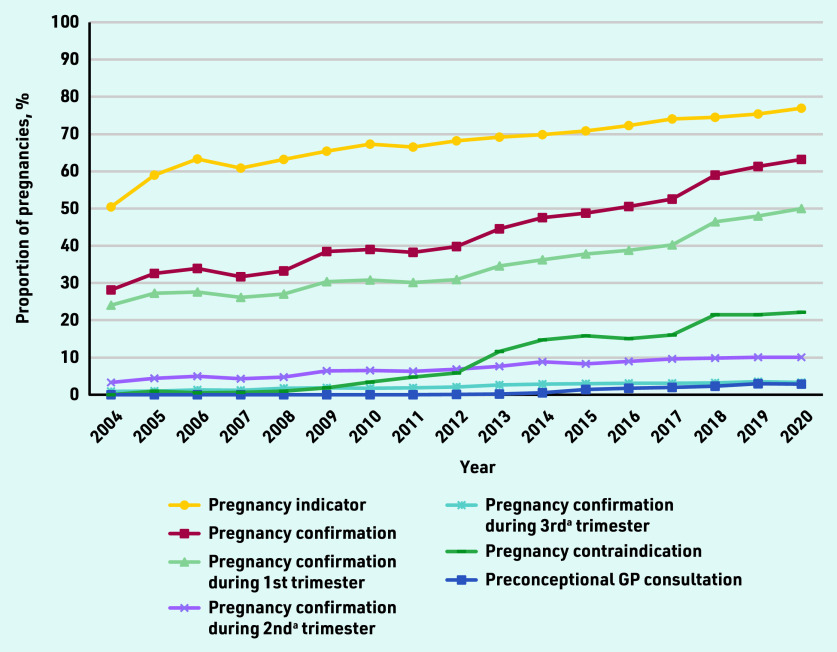
*Selected GP-recorded pregnancy indicators reflecting GPs’ awareness of pregnancy over time. ^a^ First recorded, that is, without recorded pregnancy confirmation in the prior trimester(s). All trends over time were statistically significant at* P*<0.0001.*

Figure 1 presents the GP-recorded pregnancy indicators reflecting GPs’ awareness of pregnancy over time. A strong increase was observed for the proportion of pregnancies with confirmation from 28% in 2004 to 63% in 2020 (48% overall). In total, 78% (*n* = 52 640/67 496) of these pregnancy confirmations happened first during the first trimester, then 17% (*n* = 11 426/67 496) during the second, and the remaining 5% (*n* = 3430/67 496) in the third trimester. Using all available GP records (that is, coded as well as based on search terms occurring in free text) as a pregnancy indicator, this proportion was 70% (*n* = 99 289/140 976) and increased from 50% (*n* = 61/121) in 2004 to 77% (*n* = 7025/9124) in 2020. Even though recording pregnancy as a contraindication clearly increased during the second half of the study period, such a registration was observed for only 13% (*n* = 17 643/140 976) of pregnancies. Overall, only 1% (*n* = 1626/140 976) of pregnancies were preceded by a GP consultation specifically set up and coded for preconception counselling.

### Use of hazardous medication

During 22% (*n* = 31 523/140 976) of included pregnancies GPs prescribed hazardous medication. At the point at which such hazardous medication was prescribed for the first time, pregnancy was confirmed in the GP records for only 29% (*n* = 9265/31 523). Any indicator was recorded for less than half of the women prescribed hazardous medication (*n* = 14 212/31 523, 45%). For highly hazardous medication, which was prescribed in 3% (*n* = 4489/140 976) of pregnancies, these proportions were even lower: pregnancy was confirmed for only 13% (*n* = 585/4489) and any indicator was available for 26% (*n* = 1171/4489). Comparing GP prescriptions of highly hazardous medication during pregnancy before and after pregnancy confirmation, only 11% (*n* = 127/1160) of women with such a prescription before confirmation also had such a prescription after confirmation (data not shown).

### GPs’ awareness and hazardous medication

**Figure 2.**
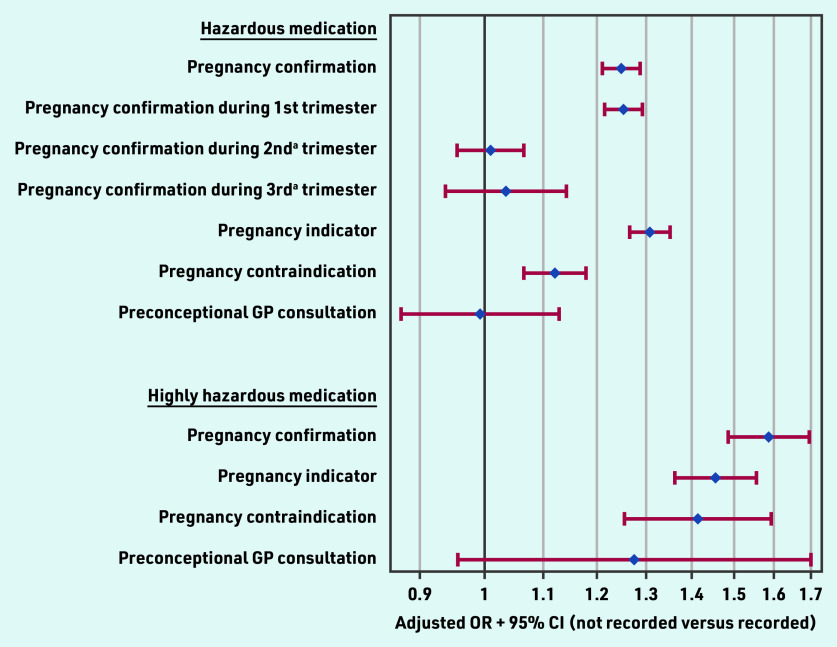
*Likelihood of (highly) hazardous medication being prescribed during pregnancy by selected GP-recorded pregnancy indicators reflecting GPs’ awareness. ^a^First recorded, that is, without recorded pregnancy confirmation in the prior trimester(s). OR = odds ratio.*

Figure 2 shows the likelihood of (highly) hazardous medication being prescribed during pregnancy by the defined pregnancy indicators. Women without a pregnancy confirmation were 25% more likely to be prescribed hazardous medication during pregnancy compared with those with confirmation, with an adjusted OR of 1.25 (95% CI = 1.21 to 1.29).

No significant association between pregnancy confirmation and prescribed hazardous medication was found if confirmation occurred for the first time during the second (OR 1.01, 95% CI = 0.96 to 1.07) or third (OR 1.04, 95% CI = 0.94 to 1.14) trimester. For highly hazardous medication, the absence of a pregnancy confirmation was associated with a 59% higher odds of prescribing highly hazardous medication (OR 1.59, 95% CI = 1.49 to 1.70). The absence of a recorded contraindication for pregnancy significantly increased the prescription of hazardous medication (OR 1.12, 95% CI = 1.07 to 1.18), particularly for highly hazardous medication including drugs actually contraindicated during pregnancy (OR 1.41, 95% CI = 1.26 to 1.59).

**Figure 3.**
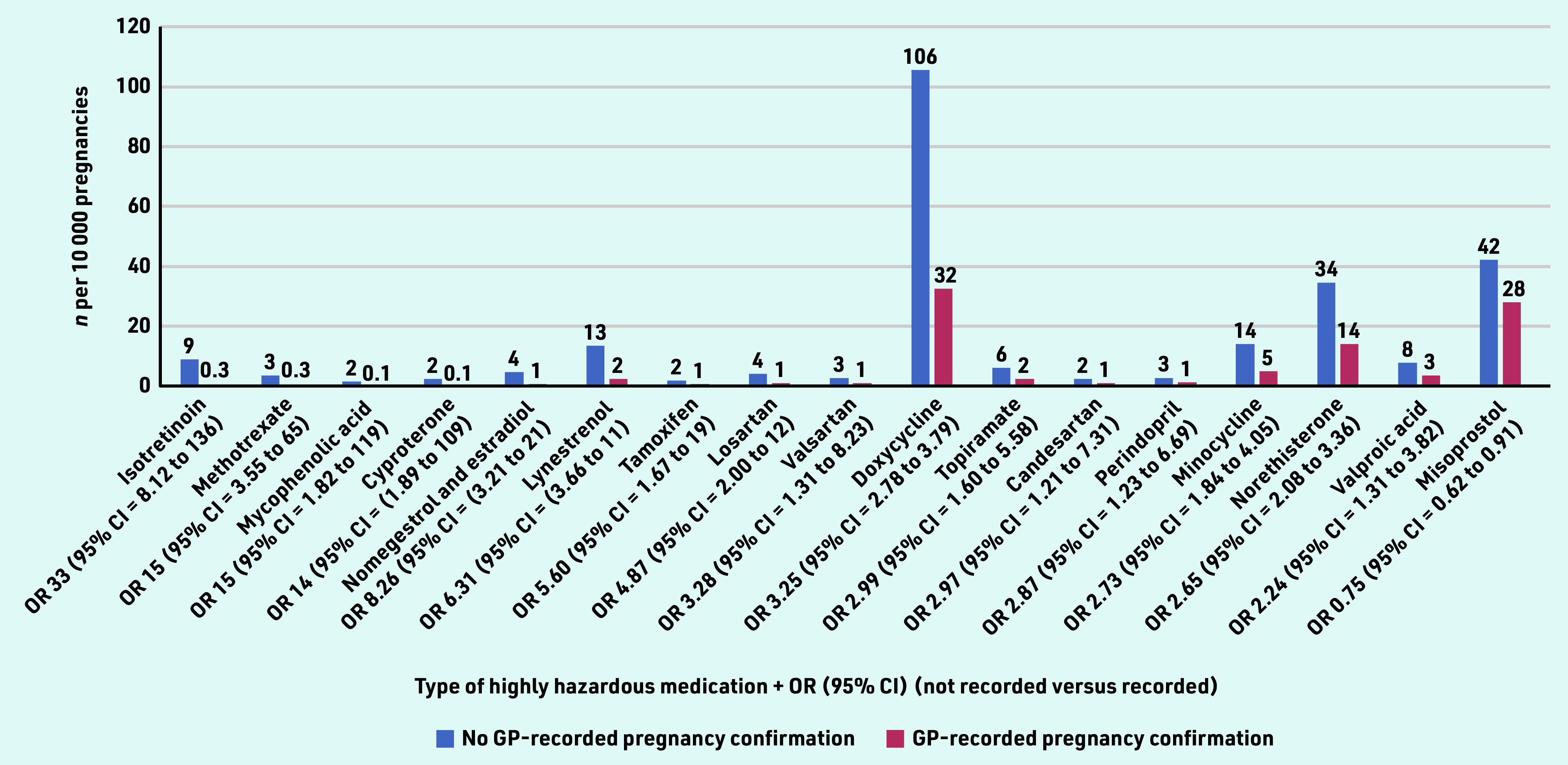
*GP prescriptions of highly hazardous medication during pregnancy, stratified by presence of a GP-recorded pregnancy confirmation. OR = odds ratio.*

Taking a closer look at the type of medication, Figure 3 presents the highly hazardous medication that was significantly more often prescribed by GPs unaware of pregnancy compared with those who were aware. Absolute numbers per drug were generally below 10 per 10 000 pregnancies, however, the top 3 relative differences were: isotretinoin (used to treat severe acne), which was prescribed about 30 times more often by GPs not aware of pregnancy, followed by methotrexate (used to treat inflammatory conditions and certain types of cancer) and mycophenolic acid (used to treat autoimmune conditions and to prevent organ rejection after transplant).

In terms of absolute prescription rates, the top 3 consisted of doxycycline (used for bacterial infections such as acne), followed by misoprostol (used to prevent stomach ulcers, but also to induce abortion), and norethisterone (used for various menstrual problems) (Figure 3).

Sensitivity analyses using medication dispensed in the pharmacy and by categorised year of delivery provided similar results for the analyses on GPs’ awareness and hazardous medication (see Supplementary Figures S1 and S2).

## DISCUSSION

### Summary

Among 140 976 selected pregnancies, for only 48% a pregnancy confirmation was recorded in the GP records, indicating GPs’ (potential) awareness of pregnancy. A statistically significant increase was observed from 28% in 2004 to 63% in 2020. The large majority (78%) of these confirmations happened during the first trimester. In 3% of all included pregnancies, the GP prescribed highly hazardous medication with teratogenic effects that should have (temporarily) been avoided, with pregnancy being GP confirmed for only 13% at the first occurrence of such a prescription. In 11% of patients with a highly hazardous prescription before pregnancy confirmation this prescription was repeated after confirmation. Comparative analyses showed that women without a GP-recorded pregnancy confirmation were 59% more likely to be prescribed this highly hazardous medication. Even though this study demonstrated that the absence of a recorded pregnancy contraindication in the GP system for automatic drug surveillance significantly increased the odds by 41% of prescribing highly hazardous medication, such an active link was created in only 13% of pregnancies (increasing from 0% to 22% during the study period). When using all available coded and uncoded electronic records from the GP information system, a pregnancy indicator was available for 70% of pregnancies. However, in 2020, 23% of pregnancies were still not registered in the GP information system. Only 1% had a GP consultation specifically set up for preconception counselling recorded in the 12 months preceding pregnancy.

### Strengths and limitations

A major strength of this study was the use of over 15 years of routinely collected data from a unique and large population- based linked cohort, shown to be representative of the Dutch population.^17^ The timing of registered records relative to pregnancy could be accurately assessed based on information from the different databases. This study thereby provides a unique, contemporary perspective on GP involvement in pregnancy in real-world clinical practice.

There are, however, several limitations. The qualitative concept of GPs’ awareness was quantified by using available electronic patient records from the women’s medical file, however, this should be regarded as ‘potential’ awareness. GPs may have been aware of pregnancy, but did not record this for a variety of reasons (for example, time constraints, deemed redundant, system difficulties, or because no letter was even received from the midwife). It has been acknowledged, however, that GPs rely on the information registered in their systems for providing accurate patient care, for example, in case of transfers to other caregivers.^27^^,^^28^

A common challenge in using administrative data is defining drug exposure or compliance. Prescription records can only approximate actual exposure and, particularly during pregnancy, prescriptions may not be filled or drugs may be discontinued. Although use of hazardous medication could therefore have been overestimated, it is not expected to have altered the conclusions in relation to GPs’ awareness, since sensitivity analyses using medication that was dispensed in the pharmacy provided similar results. Underestimated drug exposure is likely because specialist-prescribed and over-the- counter drugs were not included, however, the intended focus of the current study was on GPs’ prescription practices. Confounding by indication could not be ruled out in the definition of hazardous medication. Although pregnancy-driven prescriptions were specifically excluded where possible, some of the hazardous medication may still have been prescribed because of pregnancy. For example, as observed in the drug-level assessment, misoprostol may have been prescribed to induce abortion. However, since prescribing for these reasons would normally occur in secondary care, they are assumed to be prescribed for other indications in most cases. Unfortunately, data on the indication of use was only available for a small proportion of prescriptions. Absolute rates presented as part of the drug-level assessment should therefore be interpreted with caution and conclusions can be drawn from relative comparisons by GPs’ awareness status.

### Comparison with existing literature

This study contributes valuable new evidence to the role of GPs in daily clinical practice during pregnancy. Although existing literature on the outcomes of interest is scarce, one previous Dutch study reported a recorded diagnosis for pregnancy in the GP records for 41% of births from 2007–2009, which is very similar to what is observed in the current study.^3^ Taking into account slight differences in study period and design, the findings of prescribed (highly) hazardous medication were in agreement with previous studies.^29^^–^^32^

To the authors’ knowledge, this is the first study to assess the association between GPs’ awareness of pregnancy and prescribing hazardous medication. Although no comparison information is available on the registration of pregnancy as a contraindication in the GP information system, efforts have been made to measure the quality of GP registrations by defining a set of quality indicators, such as the proportion with a recorded contraindication. This was reported to vary among GP practices and thus they were instructed to critically review their daily habits for registering contraindications.^33^ More generally, maintaining medical records has been acknowledged as a fundamental part of a doctor’s duties in providing patient care and despite this importance, it is often given low priority.^27^^,^^28^

Similar to this study, many other previous studies have concluded that delivery of preconception care is inadequate.^11^^,^^12^^,^^34^^–^^36^ A Dutch survey conducted among GPs and midwives reported that only 0.7% of GPs systematically invited patients for a formal preconception care consultation.^11^ Although this is similar to the 1% observed in the current study, 20% of those GPs who were surveyed^11^ indicated that they performed preconception care in a standardised manner, which is probably not captured by the strict definitions used for a preconceptional GP consultation in the current study. For example, it is likely that a GP may counsel women preconceptionally as part of a consultation (coded) for something else such as the underlying condition. Although Dutch guidelines have clearly advocated standardised preconception care for some time,^34^ collaboration between GPs and other caregivers is advised. There seems to be a shift towards a more public, programmatical approach incorporated in the daily care provided that may explain the low occurrence of systematic preconceptional GP consultations observed in the current study.^37^^,^^38^

In comparison with other countries with similar healthcare systems, including the UK, similar conclusions have been drawn on the need for shared, multidisciplinary pregnancy-related care programmes in which preconception care should be offered.^10^^,^^13^^,^^14^^,^^39^

### Implications for research and practice

The finding that women without a GP-recorded pregnancy confirmation were significantly more likely to be prescribed hazardous medication indicates a potential awareness issue at the time these drugs are prescribed, placing women and their babies at avoidable risk of exposure to teratogens. Although pregnancy registration by GPs improved over the years, inadequate use still seems to be made of the available information systems for appropriate drug surveillance. The key challenge for improved registration lies with the shared responsibility, in which collaborative care is pivotal. The authors pose three main implications based on the study findings in combination with the existing evidence.

First, caregivers should be supported and educated in maintaining accurate and readily available patient records for effective communication and information transfer to other involved caregivers. Specifically in pregnancy this requires continuity of care by documenting medical records on a daily basis to prevent use of harmful medication because of delayed or incomplete record keeping.

To achieve this, the second implication relates to the electronic information systems used to maintain records. There should be clear and standardised procedures for recording and communicating information so that healthcare providers know what is expected. The differences in GPs’ awareness observed between GP information systems suggest the need for further standardisation of systems. The increased availability of pregnancy indicators when using all available records from the GP information system implies difficulties in choosing the appropriate GP reference tables for registering pregnancy, obstructing GPs’ awareness.

Further simplification would be helpful, for example, by automatically establishing an active contraindication in that specific GP reference table in case of pregnancy confirmation, blocking the prescription of certain high-risk drugs and avoiding alert fatigue among caregivers. A financial incentive was provided by the Dutch government in 2012 and 2013 for improvement of coded registration in GP practices,^40^ which is also reflected in the increased coded pregnancy indicators in the second half of the study period. During the study period the conversion of GP records from handwritten to computerised also took place.

Third, public awareness about the potential risks of medication used during pregnancy should be improved by means of population-wide education incorporating collaborative preconception care. In addition to caregivers acknowledging their duty here, this would ultimately increase women’s self-awareness recognising their own responsibility in timely informing caregivers about (planned or unplanned) pregnancy, so that appropriate action can be taken. When prescribing hazardous medication, raised awareness would make prescribers more actively enquire about pregnancy, even in the case of repeat prescriptions. Interventions should be set up in such a way that women are informed about the potential pregnancy risks of the medicines they use as early as possible, so that the patient is alert if she is considering conceiving. Whether interventions have the intended positive effects should be evaluated according to predefined targets. Pregnancy prevention programmes for highly hazardous drugs should be continuously evaluated and set up as needed.^41^

Future qualitative research among GPs, midwives, and pharmacists would be very useful to further estimate the scale of the posed awareness issue and associated aspects, such as the women’s lack of awareness of pregnancy, shortcomings of information systems, and the barriers perceived in collaborative care.

## Data

Data are available on reasonable request. Requests for sharing study data must be made on specific grounds, either 1) with the aim of corroborating the study results in the interest of public health, or 2) in the context of an audit by a competent authority. Sufficient information needs to be provided to confirm that the request is made for one of the above- mentioned purposes, including a sound justification and, in case of a request with a view to corroborate study results, a protocol on the research for which the data will be used or a plan for quality control checks, as applicable.
